# Examining Liver Cancer Patients and a High-Risk Group in a Vulnerable Area: An Experience from the Ulleung Liver Cancer Prevention and Management Project

**DOI:** 10.3390/ijerph17217757

**Published:** 2020-10-23

**Authors:** Dong-Hee Ryu, Yu-Mi Lee, Ji Mi Park, Won Young Tak, Nam-Soo Hong

**Affiliations:** 1 Department of Preventive Medicine, Catholic University of Daegu School of Medicine, Daegu 42472, Korea; ryudh@cu.ac.kr; 2 Department of Preventive Medicine, School of Medicine, Kyungpook National University, Daegu 41944, Korea; yumilee@knu.ac.kr; 3 Department of Preventive Medicine, Regional Cancer Center, Kyungpook National University Medical Center, Daegu 41404, Korea; feliz31@naver.com; 4 Department of Internal Medicine, Kyungpook National University Hospital, Daegu 41944, Korea; wytak@knu.ac.kr

**Keywords:** awareness, early detection of cancer, health behavior, liver neoplasm, residence characteristics

## Abstract

Ulleung county is a small island on the eastern side of the Korean peninsula. The Ulleung Liver Cancer Prevention and Management Project was launched in order to minimize newly developing liver cancer within this region. Population-based regional cancer registry data were analyzed to investigate the status and characteristics of registered liver cancer patients. The Interagency Workgroup of the project provided a special screening program from 1 November to 3 November 2018, and from 23 April to 25 April 2019, undertaking liver cancer screening and health behavior surveys. Logistic regression analysis was used to determine factors associated with recent liver cancer screening. In Ulleung county, hepatocellular carcinoma was identified as the main type of liver cancer, accompanied by a high incidence of hepatitis B. Approximately 25.0% of the participants were not aware of their liver condition. People who were aware of their liver condition and those who reported a general understanding of hepatitis B were more likely to have undergone recent liver cancer screening. To prevent the development and spread of the disease in the community, adequate infrastructure for cancer screening and an enhanced monitoring system are required, along with measures to create awareness to periodically determine liver condition in high-risk groups.

## 1. Introduction

Liver cancer is the sixth most commonly observed cancer type and the second major cause of death among cancer patients, following lung cancer, in Korea [1]. The National Health Insurance system in Korea ensures screening of liver cancer along with gastric, colorectal, breast, and cervical cancer. Although it is not usually recommended worldwide, the government provides financial support for liver cancer screening concerning the disease burden of the nation. The National Cancer Screening Program (NCSP) defines a high-risk group for liver cancer as those individuals who are at least 40 years old and meet one or more of the following criteria: (i) liver cirrhosis, (ii) hepatitis B virus (HBV) antigen-positive, (iii) hepatitis C virus (HCV) antibody-positive, and (iv) chronic hepatitis B or C virus infection [2]. The high-risk group is provided with the national liver cancer screening program, including a serologic test for alpha-fetoprotein and abdominal sonography, every six months. The guidelines were established on the basis of a systematic review conducted by the members of the Guideline Developing Committee [2]. The National Health Insurance Service (NHIS) leaves the high-risk group under its registration system and monitors their check-up status. 

Ulleung county comprises a small island with an area of 72.91 km^2^, located on the eastern side of the Korean peninsula [3]. According to the 2019 statistics, the registered population included 9617 individuals [3,4]. As per the National Cancer Statistics, the age-standardized liver cancer incidence (per 100,000 persons) in Ulleung county was 64.2 during 1999–2003 and 53.2 during 2009–2013 [5]. During the same period, the Korean national figures were 27.5 and 22.7, respectively, and those for Gyeongbuk province (including Ulleung county) were 30.8 and 25.3, respectively [5]. Notably, the age-standardized liver cancer incidence in Ulleung county for males has consistently been the highest among the 253 regional entities during 1999–2013 [5]. According to the 2016 National Cancer Statistics, the age-standardized liver cancer mortality rate (per 100,000 persons) in Ulleung county was 33.1 in 2016, which notably exceeded the national rate (14.3) and that of Gyeongbuk (13.9) [6]. 

The Ulleung Public Community Medical Center is responsible for the overall medical care and health services for the community. According to recent statistics, a total of 16 doctors are currently employed at the center, with most of them being public health doctors [7]. This center has been designated as the sole medical institution to provide the NCSP in Ulleung county, but it only provides a cervical cancer screening program. Shortages in examination equipment and materials, as well as irregular rotations of public health doctors, are the major obstacles in establishing an effective and sustainable screening system in the area. According to the 2016 Korea Community Health Survey, 52.7% of the sampled population in Ulleung county responded that they had not been screened for cancer in the previous two years [8]. 

To reduce the number of individuals diagnosed with liver cancer in Ulleung county, where the burden of the disease is already high, the Daegu–Gyeongbuk Regional Cancer Center launched the Ulleung Liver Cancer Prevention and Management Project in January 2018. This project aims to prevent the occurrence of the disease in the area by effective management of the registered high-risk group and to promote liver cancer screening and healthy behavior. The present study was initiated as a part of the Ulleung Liver Cancer Prevention and Management Project. The purpose of the study was to investigate the status and characteristics of registered liver cancer patients in Ulleung county using the population-based regional cancer registry (PB-RCR) and to determine factors associated with recent liver cancer screening using survey-derived data. Through this study, we expected to summarize the achievements of the Ulleung Liver Cancer Prevention and Management Project thus far.

## 2. Materials and Methods 

In this study, two types of data were used: the PB-RCR data and liver cancer high-risk group survey data. 

### 2.1. PB-RCR

#### 2.1.1. Data Collection 

The cancer incidence in Korea is calculated on the basis of the Korea Central Cancer Registry database [9]. Medical institutions equipped with a hospital-based cancer registry system and having one or more certified medical record technician(s) are classified as “registered organizations” and provide data to the Central Cancer Registration Center [9]. Medical records from medical institutions that do not have a hospital-based registry are reviewed by the Regional Cancer Centers to ensure that complete data are provided to the PB-RCRs [9]. Any cancer patient data missed through medical record reviews are obtained from National Death Report data, and anyone whose cause of death has been recorded as cancer in a death report is included in the cancer statistics [10]. The Regional Cancer Centers are responsible for collecting and managing regional cancer registries and are authorized to analyze the data confirmed by the Central Cancer Registration Center. 

The present study used the 2002–2016 PB-RCR data from the Daegu–Gyeongbuk Regional Cancer Center to investigate the status of registered liver cancer patients in Ulleung county. Patients with liver cancer were defined as persons with disease code C22 (liver and intrahepatic bile ducts) according to the International Classification of Diseases for Oncology 3rd edition (ICD-O-3) [11]. The total number of registered liver cancer patients in the Daegu–Gyeongbuk region during the period was 20,381 (8244 from Daegu (DG), 12,046 from Gyeongbuk (excluding Ulleung county) (GBE), 91 from Ulleung). The variables included in the PB-RCR data were sex, age, primary cancer site, histological diagnosis, summary stage, and survival status. The survival status was classified on the basis of the cause of death reported to Statistics Korea as of 31 November 2018, or on the date of death identified by the Ministry of Public Administration and Security. 

#### 2.1.2. Statistical Analysis of the PB-RCR Data

To compare the difference in the registered status, we calculated 5-year standardized incidence ratios (SIRs) for DG, GBE, and Ulleung county. The standardization was completed with indirect adjustment and the index populations of mid-years were used. Considering the small number of liver cancer patients in Ulleung county, we performed propensity score matching analysis to compare the characteristics of registered liver cancer patients according to residential areas. For each patient, a propensity score was calculated by logistic regression, with sex and age as covariates. The minimum distance method was used to match patients with similar propensity scores.

### 2.2. Survey Data Obtained from the High-Risk Group

#### 2.2.1. Liver Cancer Screening and Health Behavior Survey

The Regional Cancer Center organized an Interagency Workgroup to effectively implement the project. The workgroup comprised members from the Department of Health Policy Division of the Gyeongbuk local government, the Ulleung Public Community Health Center, the regional branch of the NHIS, the Liver Cancer Center of Kyungpook National University Medical Center, the Department of Preventive Medicine in Kyungpook Medical School, and the Regional Cancer Center. Considering a lack of infrastructure for liver cancer screening in Ulleung county, the Interagency Workgroup provided a special cancer screening program for the residents from 1 November to 3 November 2018 and from 23 April to 25 April 2019, undertaking liver cancer screening and health behavior surveys. The regional branches of the NHIS estimated that the numbers of the registered high-risk group among Medicaid beneficiaries and national health insurance beneficiaries within the lower 50% of national health insurance premium in Ulleung county were 174 in 2018 and 187 in 2019. As an authorized organization that can promote cancer prevention in the region, the Ulleung Public Community Medical Center officially received the list of high-risk individuals (Medicaid beneficiaries and national health insurance beneficiaries within the lower 50% of health insurance premium) from the regional branch of the NHIS. The Ulleung Public Community Medical Center staff contacted each individual on the list by phone and identified people who had not completed the national liver cancer screening but were willing to undergo special liver cancer screening. Through this process, people who were not registered on the NCSP system or registered high-risk individuals who were national health insurance beneficiaries within the higher 50% of insurance premiums were excluded. A total of 54 people voluntarily participated in the screening program and completed abdominal ultrasonography and blood tests (alpha-fetoprotein), according to NCSP guidelines. Well-trained nurses verbally explained the purpose and contents of the high-risk group survey to the participants, and informed consent was obtained from 53 participants. 

The questionnaires included questions regarding cancer screening, as required for anyone participating in the NCSP, and items related to risk perception, sociodemographic characteristics, and health-related characteristics. The questionnaires were reviewed by the members of the Interagency Workgroup first, and a second reviewer, an epidemiologist, rechecked it to ensure that it did not contain common errors. The survey protocol was reviewed and approved by the Institutional Review Board (approval no. knuch 2018-11-005). After the participants completed the questionnaires, their body mass index (BMI) was obtained using a height measurement instrument (BSM330, Biospace Inc., Seoul, Korea) and a body composition analyzer (InBody770, Biospace Inc., Seoul, Korea). 

#### 2.2.2. Study Variables

Participants who responded that they had been diagnosed with HBV infection, chronic hepatitis B, chronic hepatitis C, or liver cirrhosis were defined as people who were aware of their liver condition, while participants who responded that they had never been diagnosed with any of these conditions were defined as people who were not aware of their liver condition. Participants who responded that they understood what was involved with hepatitis B, hepatitis C, or liver cirrhosis were considered people with knowledge concerning these diseases. The sociodemographic characteristics comprised sex, age, residence duration in Ulleung, education, and monthly income. Health-related characteristics were further classified into general health behavior, risky health behavior, and a family (parents, siblings, or children) history of cancer. General health behavior variables included current smoking status, alcohol consumption (7 or more drinks for males and 5 or more drinks for females in a single session at least 2 days per week), and obesity (BMI ≥ 25 kg/m^2^). Risky health behavior variables included experiences of sharing sanitary products with others, procedures involving questionable hygiene, and past sexual behavior (sexual intercourse with non-spouse, sexual intercourse with the same sex, and diagnosis of sexually transmitted infection (STI)). The sanitary products shared with others included razors, nail clippers, and toothbrushes. Participants who reported that they had shared any of these items with others were defined as those with sharing experiences. The procedures involving questionable hygiene included tattooing, piercing, illegal drug injection, and acupuncture/cupping at non-medical facilities. Participants who reported that they had undergone any of these procedures were defined as those with such procedure experience.

The dependent variable was liver cancer screening performed within the past year. People who had undergone liver cancer screening according to NCSP guidelines (liver cancer screening within the past year through liver sonography and blood test) were considered people with liver cancer screening experience.

#### 2.2.3. Statistical Analysis of Survey Data Obtained from the High-Risk Group

For frequency analysis, we used a chi-squared test or Fisher’s exact test. To determine the associations between recent liver cancer screening and risk perception, sociodemographic characteristics, and health-related characteristics, we used logistic regression analysis. The logistic regression models were designed through adjusting sex and age. All statistical analyses were performed with SAS, version 9.4 (SAS Institute Inc., Cary, USA), and a *p*-value of <0.05 was considered indicative of statistical significance.

## 3. Results

### 3.1. Registered Liver Cancer Patients in Ulleung

Table 1 represents the 5-year SIRs for DG, GBE, and Ulleung county. For all 5-year SIRs, the values obtained for Ulleung county were higher than those for DG and GBE, but there was no significant difference. For Ulleung county, the SIRs for males were higher than those obtained for females, but the confidence intervals (CIs) overlapped in each period. While GBE showed SIRs higher than 1, those for DG have remained below 1 since 2006.

Table 2 shows the characteristics of liver cancer patients identified during 2006–2016. Of the registered patients in Ulleung, 78.0% were males and 88.0% were older than 50 years. The primary tumor site of patients in Ulleung was predominantly liver. Histologically, hepatocellular carcinoma (HCC) formed the highest proportion of all liver cancer in Ulleung county. There were significant differences in the summary stage and all-cause survival status when matched. However, there was no significant difference according to residential areas when analyzed by the cause of death. Moreover, there was a significant difference in survival duration (years) according to the residential areas when deaths caused by liver cancer were examined. The mean survival time for liver cancer patients in Ulleung county was shorter.

### 3.2. High-Risk Individuals in Ulleung

#### 3.2.1. Sociodemographic Characteristics and Risk Perception in High-Risk Individuals

Table 3 shows sociodemographic characteristics and the level of risk perception in high-risk individuals in Ulleung. Of the 53 individuals who participated in the high-risk group survey, 52.8% were males and 84.9% had resided in Ulleung county for more than 10 years (the mean residence duration of the participants was 41.0 years). Although all the participants were registered with the NCSP and were eligible for the national liver cancer screening program, 24.5% had not been aware of their liver condition. Among the participants who were aware of their liver condition, 90.0% reported that they had been identified as carriers of HBV or as patients with chronic hepatitis B (data not shown). The proportion of those with chronic hepatitis C and liver cirrhosis was 7.5% and 2.5%, respectively (data not shown). The level of understanding concerning hepatitis C was relatively lower (28.3%) than those concerning hepatitis B or liver cirrhosis (54.7% and 56.6%, respectively). Of all the participants, 58.5% reported recent liver check-ups. Among the participants who were not aware of their liver condition, only 15.4% reported liver check-ups while 72.5% who were aware of their liver condition reported having had check-ups. When adjusted for sex and age, participants who were aware of their liver condition were more likely to report recent liver check-ups than those who were not aware of their liver condition (adjusted odds ratio (aOR), 18.84; 95% CI, 2.82–126.14). In addition, participants who reported having a general understanding of hepatitis B were significantly more likely to have undergone recent liver check-ups than those without a general understanding of the disease (aOR, 8.70; 95% CI, 1.56–47.92).

#### 3.2.2. Health Behavior of High-Risk Individuals

Table 4 shows health behavior of high-risk individuals in Ulleung. Of all the participants, 15.1% and 1.9% reported sharing nail clippers and razors, respectively (1.9% reported sharing both nail clippers and razors). No participants reported sharing a toothbrush with others. It was found that 37.7% reported that they had experienced procedures involving questionable hygiene (tattooing, 28.3%; piercing, 13.2%; acupuncture/cupping at non-medical facilities, 1.9%) and 13.2% reported sexual intercourse with a non-spouse. No one reported sexual intercourse with the same sex or of a diagnosis of an STI. More than half of the participants reported a family history of cancer. Recent liver cancer screening showed negative associations with current smoking, obesity, non-exercise, having shared personal sanitary products with others, no HBV vaccination, and blood transfusion. However, there was no significant differences.

## 4. Discussion

In the present study, the 5-year SIRs were calculated, and general characteristics of liver cancer patients were analyzed using the PB-RCR data. A high liver cancer incidence in Ulleung county was further confirmed, as already shown in publicly available statistical data, using direct standardization [5]. The results also indicated that the type of liver cancer in Ulleung county was mainly HCC. In Korea, approximately 65–75% of patients with HCC have hepatitis B surface antigen (HBs Ag) positivity and 10–20% have anti-HCV positivity [12,13,14]. In the present study, 90% of the participants who were aware of their liver condition reported themselves as carriers of HBV or as patients with chronic hepatitis B (data not shown). According to a study conducted in Jindo, Jeonnam [15], the incidence of excess liver cancer in Jindo was associated with hepatitis C, indicating that the factors related to excess liver cancer incidence may differ, even among otherwise similar island regions. Although related data were not given in this study, the Interagency Workgroup of the Ulleung Liver Cancer Prevention and Management Project was able to obtain 2006–2016 NHIS medical utilization data, and by analyzing the data, it was found that there was a high medical utilization among HCC patients in Ulleung county for hepatitis B; the proportions of HCC patients diagnosed with acute or chronic hepatitis B/C were 68.8%/9.2%, while there were no autoimmune hepatitis patients. Further epidemiological studies including blood tests are required to determine any environmental or other established risk factors that are associated with a high incidence of HBV infection in the region. Some studies have shown that progression to liver cirrhosis and liver cancer can be prevented with proper hepatitis control and treatment [16,17], suggesting that the focus of the Ulleung Liver Cancer Prevention and Management Project should be on disease control and its related factors. The PB-RCR data were limited to brief information on the registered patients; hence, information concerning individual healthcare utilization and health behavior could not be obtained. However, NHIS medical utilization data lack information about the summary stage of cancer patients. If the PB-RCR data were linked to the medical utilization data held by the NHIS, then research on the relationship between cancer development and underlying diseases or health behavior would become easily accessible, and therein valuable information could be obtained. 

Liver cancer is also known to be linked to other risk factors, mainly including excessive alcohol consumption, smoking, obesity, diabetes, and autoimmune hepatitis [18,19,20]. According to the 2018 Korea National Health and Nutrition Examination Survey (KNHANES) data, the national average for obesity was 35.0% and for people who regularly exercise was 44.9% [21], while that for Ulleung county was 54.7% and 35.9%, respectively, using the high-risk survey data. Compared to the national estimates [21], rates of current smoking status and alcohol consumption of the present study showed no notable differences. By analyzing the 2006–2016 NHIS medical utilization data mentioned above, it was found that the number of HCC patients involved with medical utilization due to non-alcoholic fatty liver disease or autoimmune hepatitis was not significantly different according to residential areas. In addition, the proportion of HCC patients in Ulleung who received medical care due to diabetes was 57.0%, while that of HCC patients in Daegu was 49.5%. The results indicated that further studies that focus on the association between a high incidence of HCC and obesity-related factors in the region are required. 

Although a small number of participants were included, the present study was able to analyze the characteristics of high-risk individuals in Ulleung. It was found that approximately 25% of the participants did not know about their liver condition. An important point to be considered is that all the participants were registered under the NHIS registration system and beneficiaries of the national liver cancer screening program, which means that they met the inclusion criteria. One possible explanation for this may be the high proportion of people with a low educational level [22]. Participants who responded that they were not aware of their liver condition may experience liver diseases without knowing it, and this raises another health issue. It might explain the shorter survival time of the liver cancer patients in Ulleung presented in the study. Being aware of one’s health condition induces a person to seek management and control of the disease, and hence the deterioration of health conditions and even death can be prevented. Management of high-risk individuals should be accompanied by providing education to these individuals regarding disease control, including the necessity of regular check-ups and clinical treatment, and should not be limited to merely the registration of the number of individuals. However, the results of this study also suggest a necessity to overview a high-risk group registration system. The registration of individuals as part of a high-risk group is completed either by the individual concerned or the NHIS. A person who has been diagnosed with liver disease by a physician could submit blood test results to the NHIS. It is also possible for the NHIS to automatically register patients according to the previous two years’ medical claims, on the basis of the presumptive diagnosis in the medical records. An issue may arise if medical institutions do not change a presumptive diagnosis based on actual blood test results, making it possible for individuals who may not have a liver disease to be registered as members of a high-risk group for liver cancer and eligible to become beneficiaries of the national liver cancer screening program. Although the accuracy of participant responses in the present study cannot be confirmed since self-reported questionnaires were used, the issue concerning how high-risk group registration is undertaken needs to be addressed regardless. 

Another important finding is that participants who were aware of their liver condition showed an association with recent liver cancer screening, with statistical significance. This implies the need to determine the level of liver condition awareness in high-risk groups to reduce future liver cancer risk by leading liver cancer screening. In addition, the results that show a significant difference for liver cancer screening based on the knowledge regarding hepatitis B are very meaningful for the prevention of HCC in the region. The degree of association between liver cancer screening and these two factors was greater than other factors related to cancer screening, such as education, monthly income, and past health behavior [22]. However, since the present study was based on a cross-sectional analysis, no causal relationship between recent liver cancer screening and liver condition awareness and knowledge concerning hepatitis B could be determined, and thus caution should be exercised when interpreting the data. In other words, the possibility that liver cancer screening has aroused awareness of the liver condition cannot be neglected. 

The survey of the high-risk group had several limitations. First, considering the issue associated with high-risk group registration, the prevalence of hepatitis B and C infections among high-risk group survey participants should be examined in similar fashion to the Jindo study [15]. The study results imply a necessity for a prospective study to be conducted in this area. Second, the health behavior and characteristics of people who were registered in the high-risk group but who did not participate in the survey could not be investigated. When identifying people who were willing to participate in the special liver cancer screening program, the Ulleung Public Community Medical Center staff tried to investigate the reasons for non-participation. Some of the identified reasons for non-participation included having completed liver cancer screening in medical institutions in the mainland, being hospitalized for other diseases, and staying at nursing facilities. Third, there is a possibility of sampling error due to volunteer bias. The study only included Medicaid beneficiaries and national health insurance beneficiaries within the lower 50% of the insurance premium. Therefore, high-risk individuals with better socioeconomic status were not evaluated. Fourth, even though some people who have been living on the island for their entire lives were included, the relationship between environmental effects and liver disease could not be determined through the study. Therefore, further investigation involving a greater number of participants should be undertaken in the future. 

Despite these limitations, the present study is the first to have examined the characteristics and status of the registered liver cancer patients in Ulleung using the PB-RCR data and determined factors associated with recent liver cancer screening using survey-derived data. 

For the Ulleung Liver Cancer Prevention and Management Project to operate more effectively, the incidence of newly diagnosed liver cancer in this area should be minimized through focused high-risk group management. According to the 2016 National Health Screening Statistical Yearbook, the national average for people undergoing liver cancer screening was 64.0%, while that for Ulleung county was only 51.0% in 2016 [23]. Cancer screening programs dealing with high-risk groups, such as those at risk of liver cancer, should be accompanied by a thorough monitoring and management system for registered individuals [24]. 

## 5. Conclusions

The following measurements need to be undertaken in order to encourage a high-risk group in a vulnerable area to perform liver cancer screening regularly. First, adequate infrastructure for cancer screening, including public health doctors subject to regular rotation, is necessary. With the help of this project, a male participant was diagnosed with liver cancer at an early stage (the abdominal ultrasonography and blood test results for 52 individuals were insignificant). Second, an enhanced NCSP monitoring system that updates the screening status of individuals quickly for more effective management of high-risk groups is required. Finally, these should also be accompanied by efforts to improve the level of understanding in the community concerning cancer screening. On the basis of the results of the present study, future strategies for high-risk groups in vulnerable areas need to be reconsidered, taking into account the liver condition awareness. Education of high-risk groups regarding disease management through online education, customized consultation, and target-specific screening promotion strategies might be helpful. 

## Figures and Tables

**Table 1:** Five-year standardized incidence ratios (SIRs) for Daegu, Gyeongbuk (excluding Ulleung), and Ulleung county (2006–2016).

Year	Region	Number of Registered Liver Cancer Patients	Total	Male	Female
2002–2006	DG	3699	1.03 (0.95–1.10)	1.04 (0.96–1.13)	1.03 (0.89–1.19)
	GBE	5505	1.10 (1.04–1.17)	1.13 (1.06–1.21)	1.04 (0.92–1.16)
	Ulleung	43	1.94 (0.86–3.45)	1.91 (0.73–3.64)	1.80 (0.17–5.15)
2007–2011	DG	3847	0.97 (0.91–1.04)	0.98 (0.90–1.06)	0.99 (0.86–1.14)
	GBE	5531	1.07 (1.00–1.13)	1.10 (1.03–1.18)	0.98 (0.87–1.10)
	Ulleung	43	1.75 (0.77–3.12)	1.72 (0.66–3.28)	1.60 (0.15–4.60)
2012–2016	DG	3621	0.92 (0.86–0.99)	0.95 (0.87–1.03)	0.90 (0.77–1.04)
	GBE	5396	1.05 (0.99–1.12)	1.08 (1.01–1.16)	0.99 (0.87–1.11)
	Ulleung	46	1.96 (0.90–3.43)	1.94 (0.78–3.60)	1.72 (0.16–4.93)

Values are presented as a 5-year standardized incidence ratio (95% confidence interval). The standardization was completed with indirect adjustment, and the index populations of mid-years were used. Abbreviations: DG, Daegu; GBE, Gyeongbuk (excluding Ulleung).

**Table 2:** Characteristics of patients diagnosed with malignant neoplasm of the liver and intrahepatic bile ducts (C22) (2006–2016).

Category	Unmatched				Matched ^*^			
	DG	GBE	Ulleung	*p*	DG	GBE	Ulleung	*p*
Total	8244	12,046	91		91	91	91	
Sex								
Male	6187 (75.1)	9018 (74.9)	71 (78.0)	0.76	65 (71.4)	69 (75.8)	71 (78.0)	0.58
Female	2057 (24.9)	3028 (25.1)	20 (22.0)		26 (28.6)	22 (24.2)	20 (22.0)	
Age								
<50	1357 (16.5)	1562 (13.0)	11 (12.1)	<0.01	23 (25.3)	22 (24.2)	11 (12.1)	0.27
50–59	2440 (29.6)	3012 (25.0)	28 (30.8)		21 (23.1)	20 (22.0)	28 (30.8)	
60–69	2193 (26.6)	3210 (26.7)	30 (33.0)		27 (29.7)	24 (26.4)	30 (33.0)	
≥70	2254 (27.3)	4262 (35.4)	22 (24.2)		20 (22.0)	25 (27.5)	22 (24.2)	
Primary tumor site ^1^								
Liver	6740 (81.8)	9412 (78.1)	78 (85.7)	<0.01	75 (82.4)	73 (80.2)	78 (85.7)	0.61
Intrahepatic bile duct	1504 (18.2)	2634 (21.9)	13 (14.3)		16 (17.6)	18 (19.8)	13 (14.3)	
Histological diagnosis ^1^								
HCC	6419 (77.9)	8845 (73.4)	78 (85.7)	<0.01	71 (78.0)	71 (78.0)	78 (85.7)	0.46
CC	1115 (13.5)	1675 (13.9)	8 (8.8)		5 (5.5)	4 (4.4)	8 (8.8)	
Combined HCC–CC	82 (1.0)	87 (0.7)	1 (1.1)		2 (2.2)	2 (2.2)	1 (1.1)	
Klatskin tumor	201 (2.4)	456 (3.8)	1 (1.1)		5 (5.5)	9 (9.9)	1 (1.1)	
Adenocarcinoma	103 (1.3)	228 (1.9)	2 (2.2)		2 (2.2)	0 (0.0)	2 (2.2)	
Neoplasm	213 (2.6)	572 (4.8)	0 (0.0)		4 (4.4)	5 (5.5)	0 (0.0)	
Other: liver or intrahepatic duct	75 (0.9)	119 (1.0)	1 (1.1)		1 (1.1)	0 (0.0)	1 (1.1)	
Other: other organs	36 (0.4)	64 (0.5)	0 (0.0)		1 (1.1)	0 (0.0)	0 (0.0)	
Summary stage								
Localized	4531 (55.0)	5363 (44.5)	48 (52.8)	<0.01	47 (51.7)	34 (37.4)	48 (52.8)	0.04
Regional	1748 (21.2)	2874 (23.9)	23 (25.3)		23 (25.3)	25 (27.5)	23 (25.3)	
Distant	1186 (14.4)	1790 (14.9)	10 (11.0)		12 (13.2)	8 (8.8)	10 (11.0)	
Unknown	779 (9.5)	2019 (16.8)	10 (11.0)		9 (9.9)	24 (26.4)	10 (11.0)	
All-cause survival status ^2^								
Survived	2119 (25.7)	2808 (23.3)	28 (30.8)	<0.01	5 (5.5)	14 (15.4)	28 (30.8)	<0.01
Deceased	6125 (74.3)	9238 (76.7)	63 (69.2)		86 (94.5)	77 (84.6)	63 (69.2)	
Cause of death (deceased only)								
Malignant neoplasm of liver and intrahepatic bile duct	5103 (83.3)	7399 (80.1)	46 (73.0)	<0.01	74 (86.1)	62 (80.5)	46 (73.0)	0.14
Others	1022 (16.7)	1839 (19.9)	17 (27.0)		12 (14.0)	15 (19.5)	17 (27.0)	
Survival time^3^								
Mean ± SD (years)	7.3 ± 22.1	6.8 ± 10.9	5.7 ± 2.9	0.26	11.2 ± 1.9	11.2 ± 2.4	5.7 ± 2.9	<0.01

Values are presented as number (%). ^*^ Propensity score matching analysis was performed. Logistic regression was performed with sex and age as covariates, and the minimum distance method was used to match patients with similar scores. ^1^ Primary tumor site and histological diagnosis were classified by the code ICD-O-3. ^2^ Survival status: as of 30 November 2018. ^3^ Survival time: survival duration (years) of patients whose cause of death was malignant neoplasm of the liver and intrahepatic bile duct (C22). Abbreviations: DG, Daegu; GBE, Gyeongbuk (excluding Ulleung); HCC, hepatocellular carcinoma; CC, cholangiocarcinoma; SD, standard deviation.

**Table 3:** Sociodemographic characteristics and risk perception in high-risk individuals in Ulleung (*n* = 53).

Category	Subgroup	Total		Liver Cancer Screening Within the Past Year				
		*n*	%	*n*	%	*p*	Crude OR (95% CI)	Adjusted * OR (95% CI)
Total		53	100.0	31	58.5	-	-	-
Sociodemographic characteristics								
Sex	Male	28	52.8	14	50.0	0.18	0.47 (0.15–1.44)	-
	Female	25	47.2	17	68.0		Ref	-
Age	<50	13	24.5	5	38.5	0.27	0.31 (0.05–1.85)	-
	50–59	13	24.5	7	53.9		0.58 (0.10–3.40)	-
	60–69	18	34.0	13	72.2		1.30 (0.23–7.32)	-
	≥70	9	17.0	6	66.7		Ref	-
Residence duration in Ulleung	<10 years	8	15.1	5	62.5	0.99	Ref	Ref
	≥10 years	45	84.9	26	57.8		0.82 (0.17–3.86)	0.26 (0.03–1.98)
Education	≤Elementary	15	28.3	12	80.0	0.04	1.33 (0.17–10.25)	
	Jr. high school	10	18.9	6	60.0		0.50 (0.07–3.85)	
	High school	20	37.7	7	35.0		0.18 (0.03–1.14)	
	≥College	8	15.1	6	75.0		Ref	
Monthly income (1000 KRW)	<500	12	22.6	8	66.7	0.04	1.50 (0.22–10.22)	0.38 (0.03–4.43)
	500–1000	10	18.9	7	70.0		1.75 (0.23–13.16)	0.55 (0.05–6.09)
	1000–2000	13	24.5	10	76.9		2.50 (0.35–18.04)	1.50 (0.18–12.76)
	2000–3000	11	20.8	2	18.2		0.17 (0.02–1.42)	0.08 (0.01–0.92)
	≥3000	7	13.2	4	57.1		Ref	Ref
Risk perception								
Liver condition awareness	Yes	40	75.5	29	72.5	<0.01	14.50 (2.76–76.15)	18.84 (2.82–126.14)
	No	13	24.5	2	15.4		Ref	Ref
General understanding about	Hepatitis B (Yes)	29	54.7	20	69.0	0.09	2.63 (0.85–8.08)	8.70 (1.56–47.92)
	Hepatitis B (No)	24	45.3	11	45.8		Ref	Ref
	Hepatitis C (Yes)	15	28.3	9	60.0	0.89	1.09 (0.32–3.69)	1.40 (0.37–5.26)
	Hepatitis C (No)	38	71.7	22	57.9		Ref	Ref
	Liver cirrhosis (Yes)	30	56.6	17	56.7	0.76	0.84 (0.28–2.54)	1.06 (0.29–3.93)
	Liver cirrhosis (No)	23	43.4	14	60.9		Ref	Ref

* Adjusted for sex and age. Abbreviations: OR, odds ratio; CI, confidence interval; Ref, reference; KRW, the currency of Korea (KRW 1000 is approximately USD 1).

**Table 4:** Health behaviors of high-risk individuals in Ulleung (*n* = 53).

Category	Subgroup	Total		Liver Cancer Screening within the Past Year				
		*n*	%	*n*	%	*p*	Crude OR (95% CI)	Adjusted * OR (95% CI)
Total		53	100.0	31	58.5	-	-	-
Current health behaviors								
Current smoking	Yes	13	24.5	4	30.8	0.02	0.21 (0.06–0.83)	0.26 (0.05–1.47)
	No	40	75.5	27	67.5		Ref	Ref
Heavy alcohol consumption	Yes	11	20.8	6	54.6	0.99	0.82 (0.21–3.11)	1.12 (0.26–4.83)
	No	42	79.3	25	59.5		Ref	Ref
BMI	≥25	29	54.7	17	58.6	0.98	1.01 (0.34–3.03)	0.93 (0.29–2.94)
	<25	24	45.3	14	58.3		Ref	Ref
Moderate exercise	No	34	64.2	18	52.9	0.27	0.52 (0.16–1.69)	0.39 (0.10–1.50)
	Yes	19	35.9	13	68.4		Ref	Ref
Risky health behaviors								
Sharing personal sanitary products with others ^1^	Yes	8	15.1	4	50.0	0.70	0.67 (0.15–3.01)	0.39 (0.07–2.13)
	No	45	84.9	27	60.0		Ref	Ref
Procedures involving questionable hygiene ^2^	Yes	20	37.7	14	70.0	0.19	2.20 (0.68–7.11)	1.68 (0.42–6.79)
	No	33	62.3	17	51.5		Ref	Ref
Intercourse with non-spouse	Yes	7	13.2	5	71.4	0.69	1.92 (0.34–10.96)	4.55 (0.60–34.75)
	No	46	86.8	26	56.5		Ref	Ref
Medical history								
HBV vaccination	No ^3^	33	62.3	19	57.6	0.86	0.91 (0.29–2.80)	0.87 (0.26–2.87)
	Yes	20	37.7	12	60.0		Ref	Ref
Diagnosis of	Cancer	2	3.8	2	100.0	0.51	-	-
	STI	0	0.0	-	-	-	-	-
Medical procedure	Blood transfusion	5	9.4	3	60.0	0.99	1.07 (0.16–7.01)	0.87 (0.12–6.49)
	Dialysis	0	0.0	-	-	-	-	-
Family history of	Cancer ^4^	28	52.8	17	60.7	0.72	1.21 (0.41–3.63)	1.48 (0.44–4.99)

* Adjusted for sex and age. ^1^ Sharing personal sanitary products (razor, nail clipper, toothbrush) with others: sharing razor, 1 (1.9%); sharing nail clipper, 8 (15.1%); sharing both razor and nail clipper, 1 (1.9%); sharing toothbrush, 0 (0.0%). ^2^ Procedures with questionable hygiene (tattooing, piercing, illegal drug injection, acupuncture/cupping at non-medical facilities): tattooing, 15 (28.3%); piercing, 7 (13.2%); both tattooing and piercing, 3 (5.7%); illegal drug injection, 0 (0.0%); acupuncture/cupping at non-medical facilities, 1 (1.9%). ^3^ Included four response failures. ^4^ Family members include parents, siblings, and children. Abbreviations: OR, odds ratio; CI, confidence interval; Ref, reference; BMI, body mass index; HBV, hepatitis B virus; STI, sexually transmitted infection.
